# An intracellular targeted antibody detects EGFR as an independent prognostic factor in ovarian carcinomas

**DOI:** 10.1186/1471-2407-11-294

**Published:** 2011-07-14

**Authors:** Aurelia Noske, Michael Schwabe, Wilko Weichert, Silvia Darb-Esfahani, Ann-Christin Buckendahl, Jalid Sehouli, Elena I Braicu, Jan Budczies, Manfred Dietel, Carsten Denkert

**Affiliations:** 1 Institute of Pathology, University Hospital Charité Berlin, Germany; 2 Institute of Pathology, University Hospital Zurich, Switzerland; 3 Institute of Pathology and National Center for Tumor Diseases, Ruprecht-Karls-Universität, Heidelberg, Germany; 4 Department of Gynecology, University Hospital Charité Berlin, Germany

**Keywords:** EGFR, CRM1, COX-2, ovarian cancer, prognosis

## Abstract

**Background:**

In ovarian cancer, the reported rate of EGFR expression varies between 4-70% depending on assessment method and data on patient outcome are conflicting. Methods: In this study we investigated EGFR expression and its prognostic value in a cohort of 121 invasive ovarian carcinomas, using a novel antibody against the intracellular domain of the receptor. We further evaluated an association between EGFR, the nuclear transporter CRM1 as well as COX-2. Furthermore, we evaluated EGFR expression in ten ovarian cancer cell lines and incubated cancer cells with Leptomycin B, a CRM1 specific inhibitor.

**Results:**

We observed a membranous and cytoplasmic EGFR expression in 36.4% and 64% of ovarian carcinomas, respectively. Membranous EGFR was an independent prognostic factor for poor overall survival in ovarian cancer patients (HR 2.7, CI 1.1-6.4, p = 0.02) which was also found in the serous subtype (HR 4.6, CI 1.6-13.4, p = 0.004). We further observed a significant association of EGFR with COX-2 and nuclear CRM1 expression (chi-square test for trends, p = 0.006 and p = 0.013, respectively). In addition, combined membranous EGFR/COX-2 expression was significantly related to unfavorable overall survival (HR 7.2, CI 2.3-22.1, p = 0.001).

In cell culture, we observed a suppression of EGFR protein levels after exposure to Leptomycin B in OVCAR-3 and SKOV-3 cells.

**Conclusions:**

Our results suggest that the EGFR/COX-2/CRM1 interaction might be involved in progression of ovarian cancer and patient prognosis. Hence, it is an interesting anti-cancer target for a combination therapy. Further studies will also be needed to investigate whether EGFR is also predictive for benefit from EGFR targeted therapies.

## Background

Epithelial ovarian cancer, commonly diagnosed in an advanced stage, has the highest mortality among gynecological malignancies [1]. Surgical tumor debulking followed by chemotherapy with a combination of platinum-taxane as first line is the currently established therapy. However, tumor relapse and development of drug resistance are major problems in this disease, and new molecular targeted therapies are urgently needed, some of which have already entered clinical trials.

Epidermal growth factor receptor (EGFR) is one of such attractive targets for anticancer therapy. Anti-EGFR drugs, like monoclonal antibodies or small molecule tyrosine kinase inhibitors have emerged as effective agents in treating metastatic colorectal cancer and non-small cell lung cancer. In contrast, clinical studies with EGFR blocking drugs in advanced ovarian cancers have shown only limited efficacy [2] but in the majority of these trials, EGFR positivity was not analyzed as a selection criterion. To date, the exact frequency of EGFR expression in ovarian cancer is not clear. The reported range of EGFR expression varies between 4-70% and is caused by different assessment methods and study cohorts [3]. Mutations are rare (only 4%), high gene copy numbers account approximately 15% [4-7], and protein over-expression is detected in up to 60% [8].

The mechanisms of EGFR activation and particularly the intracellular transactivation in ovarian cancer are not yet fully elucidated. Transduction of EGFR signals is mainly mediated by the RAS/MAPK- and PI3K/AKT-pathway [2] and shuttle proteins confer signals from the cytoplasm to the nucleus to transcription factors. CRM1 (chromosomal region maintenance/exportin 1) is an important nuclear export receptor [9] which controls shuttling of relevant tumor pathway elements like p53 [10], AKT1 [11], Her2 [12], and EGFR [13]. Furthermore, CRM1 is expressed in ovarian carcinomas, and expression is associated with prognosis as well as COX-2 regulation [14]. Therefore, we explored the possibility of an interaction between EGFR, COX-2 and CRM1 in ovarian cancer.

In this study, we investigate the EGFR protein expression using a novel antibody against the intracellular domain of the receptor in a cohort of primary invasive ovarian carcinomas as well as human ovarian cancer cell lines. We further compare expression data with clinico-pathological characteristics, patient survival as well as CRM1 and COX-2 expression. To evaluate a possible EGFR/CRM1 interaction, we incubate ovarian cancer cells with Leptomycin B, a specific CRM1 inhibitor.

## Methods

### Study population

Invasive ovarian carcinomas of 121 patients who were diagnosed at the Institute of Pathology Charité University Hospital Berlin (Germany) were included in this study, which was conducted in the framework of the tumor bank ovarian cancer (TOC) network http://www.toc-network.de. All clinical data, including all surgical procedures are documented in detail using a systematic documentation tool [15]. These protocols have been approved by the institutional review board of the Charité Hospital. Formalin fixed and paraffin embedded tumor tissue samples were evaluated on hematoxylin and eosin sections. The stage of tumors was assessed according to the International Federation of Gynecology and Obstetrics (FIGO). Silverberg Grading System was done by evaluation of architecture, nuclear polymorphism and mitotic rate [16]. The median follow-up time of the surviving patients was 38.1 months. Data on adjuvant chemotherapy was known for 113 patients. In the majority (94%), a platinum-based combination therapy was administered.

### Immunohistochemistry

Immunohistochemistry was performed on tissue microarrays (TMAs) as described previously [17]. Briefly, slides were deparaffinized and boiled in citrate buffer in a pressure cooker for 5 minutes, incubated with the monoclonal rabbit antibody directed against the internal domain of EGFR (clone 5b7, Ventana Medical Systems, Tucson, Arizona, USA). Further staining was carried out on the BenchMark XT (Ventana Medical Systems, Tucson, AZ) according to the manufactures' instructions (protocol nr. 31). EGFR expression was scored as positive if tumor cells displayed immunoreactivity in > 1% according to the scoring system used in several previously published studies [4,7,18]. Staining was evaluated by an experienced pathologist (M.S.) who was blinded towards patient characteristics and outcome. Negative control was performed by omitting the primary antibody. The antibody specifity was evaluated by Western blotting. In addition, immunohistochemical data on CRM1 and COX-2 expression was available for 60 and 62 ovarian cancer specimens from previous studies [14,19].

### Cell culture, inhibitor and immunoblotting

The human ovarian cancer cell lines (OVCAR-3, SKOV-3, ES-2, OAW42, CAOV-3, A27/80, FU-OV-1, EFO-21, EFO-27, Mdah2774) and immortalized normal human ovarian surface epithelial (HOSE) cells investigated in this study have recently been described [14,17]. Cells were incubated with Leptomycin B (LMB), a specific CRM1 inhibitor (L2913; Sigma Chemical Company) for a maximum of 72 hours at different concentrations (2.5, and 5 ng/ml) as reported previously [14].

For protein analysis, cells were lysed in 100 μl of 62.5 mM Tris-HCl (pH 6.8) containing 2% sodium dodecyl sulfate, 10% glycerol, 50 mM DTT and 0.1% bromophenole blue. 100 μg protein/sample were separated on a 10% polyacrylamide gel, blotted onto nitrocellulose membranes (Schleicher&Schuell, Dassel, Germany), washed in PBS, and blocked in buffer (1 × PBS, 0.1% Tween-20, 5% i-block (Tropix, Bedford, MA, USA)) for one hour at room temperature. Membranes were probed with the monoclonal anti-EGFR antibody (clone 5b7, Ventana Medical Systems, Tucson, Arizona, USA) overnight at 4°C, diluted 1:1000 in blocking buffer, and followed by incubation with alkaline phosphatase-conjugated goat anti-rabbit secondary antibody (Tropix). Bands were visualized using the CDP star RTU luminescence system (Tropix).

### Statistics

For statistical analysis, the SPSS software package (IBM SPSS statistics version 19.0) was used. Association of EGFR with clinico-pathological parameters was assessed by the two-sided Fisher's exact test or a chi-square test for trends as indicated. Spearman's correlation was used to evaluate an association between CRM1 and COX-2. The univariate survival analysis was done using the Kaplan-Meier method, survival curves were compared with the log rank test. Cox proportional hazard models were fitted in order to calculate hazard ratios and to carry out multivariate survival analyses. In general, p-values < 0.05 were considered significant.

## Results

### Clinico-pathological characteristics of ovarian cancer patients

Primary invasive ovarian carcinomas of 121 women were investigated for EGFR expression. Mean patient age at the time of surgery was 57 years, ranging from 33 to 80. The main histological subtype of the invasive carcinomas was serous cancer (66.1%). The group of non-serous cancer (24%) consists of the endometrioid subtype (n = 12, 9.9%), transitional cell type (n = 8, 6.6%), mucinous type (n = 5, 4.1%), and clear cell carcinomas (n = 4, 3.3%). Characteristics of the study population are given in Table 1.

**Table 1:** Characteristics of 121 patients with invasive ovarian carcinomas

Parameter	All cases (%)
**Age at surgery (years)**	
< 60	73 (60.3)
≥ 60	48 (39.7)
**Histological type**	
serous	80 (66.1)
non-serous	29 (24.0)
undifferentiated	12 (9.9)
**FIGO stage**	
I	18 (14.9)
II	10 (8.3)
III	84 (69.4)
IV	9 (7.4)
**Tumor stage**	
pT1	21 (17.3)
pT2	12 (10.0)
pT3	88 (72.7)
**Nodal stage (n = 96)**	
pN0	46 (47.9)
pN1	50 (52.1)
**Tumor grade (Silverberg)**	
G1	19 (15.7)
G2	50 (41.3)
G3	52 (43.0)
**Intraoperative residual tumor (n = 88)**	
residual tumor < 2 cm	78 (88.6)
residual tumor ≥ 2 cm	10 (11.4)
**Chemotherapy (n = 113)**	
Platinum-based	106 (93.8)
non-platinum	3 (2.7)
no chemotherapy	4 (3.5)

### EGFR expression in ovarian carcinomas

**Figure 1:**
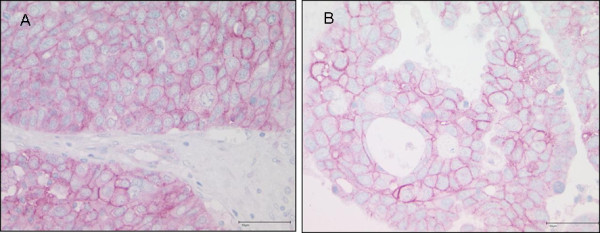
**Immunohistochemical analysis of EGFR**. A: Membranous and weak cytoplasmic expression in a high-grade serous ovarian carcinoma. B: Pure membranous expression in a high-grade serous carcinoma.

Immunohistochemical analysis was performed on 121 ovarian carcinomas (Figure 1). We observed a membranous as well as a cytoplasmic EGFR expression. Therefore both staining patterns were evaluated separately to identify a possible cell specific localisation of EGFR and to evaluate an association of a specific pattern with prognostic pathological parameters. Membrane staining is defined as any immunoreaction in part of the cell membrane (complete or incomplete), while the cytoplasmic immunoreaction is confined to the intracellular compartment. A positive membranous staining was found in 36.4% (44 out of 121) and a cytoplasmic reaction in 67% (81 out of 121). Both expression patterns were significantly associated (chi-square test for trends, p = 0.003). No associations between membranous or cytoplasmic EGFR expression, and clinico-pathological parameters as listed in Table 1 were found.

### Membranous EGFR expression is related to CRM1 and COX-2 expression

To identify an association of EGFR with CRM1 and COX-2, we compared EGFR protein expression with data on CRM1 and COX-2 expression which was investigated previously [14]. The nuclear export protein CRM1 is expressed both in the nucleus and cytoplasm of ovarian carcinomas as described recently [14]. Here, we found that membranous EGFR expression is associated with nuclear CRM1 expression (chi-square test for trends, p = 0.013). Further, membranous EGFR expression is associated with COX-2 expression (chi-square test for trends, p = 0.006). CRM1 and COX-2 expression correlated with each other (spearman correlation coefficient 0.371, p = 0.001), as described previously [14].

### Membranous EGFR expression is an independent prognostic factor for overall survival

**Figure 2:**
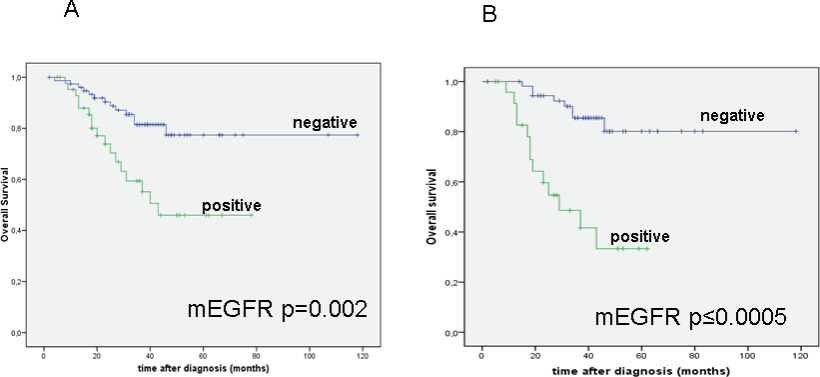
**Univariate survival analyses for membranous EGFR**. A: In Kaplan-Meier analysis, membranous EGFR is associated with poor overall survival in all ovarian carcinomas. B: Membranous EGFR is associated with poor overall survival in the subgroup of serous ovarian carcinomas.

In univariate Kaplan-Meier survival analysis and logistic regression analysis (Table 2), high tumor grade, intraoperative residual tumor, and patient age (> 60 yrs.) are significantly associated with unfavourable patient overall survival. FIGO stage, tumor and nodal stage did not reach prognostic significance in our cohort. Further, membranous EGFR expression was significantly associated with shorter overall survival (log rank, p = 0.002; Figure 2A). In a multivariate Cox regression analysis adjusted for other prognostic factors, membranous EGFR expression was confirmed as an independent prognostic factor for overall survival in ovarian cancer patients (Table 3). For cytoplasmic EGFR expression, no significant differences were found. In a subgroup analysis (Table 4), membranous EGFR expression was observed in 25 out of 80 (31.3%) serous ovarian carcinomas and significantly related to poor overall survival in this cancer subtype (log rank, p < 0.001; Figure 2B), which was confirmed in a multivariate analysis (HR 4.6, CI 1.6-13.4, p = 0.004). No association between membranous EGFR and non-serous cancer type was observed (log rank, p = 0.72). Regarding the progression-free survival, no significant differences for membranous or cytoplasmic EGFR expression (log rank, p = 0.58 and p = 0.85, resp.) were found.

**Table 2:** Univariate analysis: factors predicting overall survival

Parameter	n (%)	Overall survival	
		p-value (log rank)	HR (CI), p-value
**Membranous EGFR**	121 (100)	0.002	2.8 (1.4-5.6), 0.004
positive	44 (36.4)		
negative	77 (63.6)		
**Cytoplasmic EGFR**	121 (100)	0.79	0.9 (0.4-1.8), 0.79
positive	81 (66.9)		
negative	40 (33.1)		
**Age at surgery (years)**	121 (100)	0.003	2.8 (1.3-5.7), 0.004
< 60	73 (60.3)		
≥ 60	48 (39.7)		
**Histological type**	121 (100)	0.71	1.1 (0.7-1.9), 0.68
serous	80 (66.1)		
non-serous	29 (24.0)		
undifferentiated	12 (9.9)		
**FIGO stage**	121 (100)	0.44	1.5 (0.9-2.5), 0.13
I	18 (14.9)		
II	10 (8.3)		
III	84 (69.4)		
IV	9 (7.4)		
**Tumor stage**	121 (100)	0.14	1.6 (0.9-2.9), 0.08
pT1	21 (17.3)		
pT2	12 (10.0)		
pT3	88 (72.7)		
**Nodal stage**	96 (100)	0.05	2.7 (0.9-7.8), 0.06
pN0	46 (47.9)		
pN1	50 (52.1)		
**Tumor grade**	121 (100)	0.035	2.0 (1.1-3.5), 0.013
G1	19 (15.7)		
G2	50 (41.3)		
G3	52 (43.0)		
**Intraoperative residual tumor**	88 (100)	0.0001	5.0 (1.9-13.0), 0.001
residual tumor < 2 cm	78 (88.6)		
residual tumor ≥ 2 cm	10 (11.4)		

**Table 3:** Multivariate logistic regression analysis

Characteristic	Hazard Ratio (95% CI)	p-value
Membranous EGFR (pos. vs. neg.)	2.7 (1.1-6.4)	0.02
Age	1.1 (1.0-1.1)	0.01
FIGO stage (I+II vs. III+IV)	3.1 (0.4-23.8)	0.3
Tumor grade (G1 vs. G2+3)	1.5 (0.2-11.6)	0.7
Residual tumor (< 2 vs. ≥ 2 cm)	4.0 (1.4-11.1)	0.008

**Table 4:** Univariate survival analysis for the complete study cohort and the serous subtype

characteristic	all histological subtypes			serous subtype		
	n	Hazard ratio (CI 95%)	p-value	n	Hazard ratio (CI 95%)	p-value
**mEGFR**	120	2.8 (1.4-5.6)	0.004	77	5.8 (2.4-14.1)	< 0.001
**CRM1**	60	3.9 (1.1-14.0)	0.031	39	3.4 (0.7-17.0)	0.13
**COX-2**	62	4.9 (1.8-12.7)	0.001	41	4.8 (1.3-17.8)	0.02
**mEGFR+COX-2**	62	7.2 (2.3-22.1)	0.001	41	5.9 (1.5-23.7)	0.013

**Figure 3:**
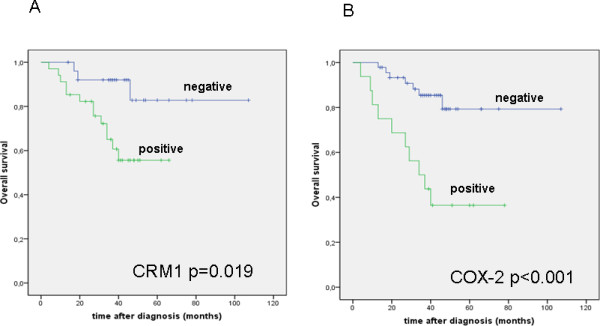
**Univariate survival analyses for CRM1 and COX-2**. A-B: CRM1 and COX-2 expression is related to unfavorable overall survival in ovarian cancer patients.

Since expression data of membranous EGFR (mEGFR) was strongly related to CRM1 and COX-2 expression levels, we aimed to evaluate the impact of a combination of these molecules on patient survival. Here, we observed that nuclear CRM1 (Figure 3A) as well as COX-2 (Figure 3B) expression is related to poor overall survival in ovarian carcinomas (log rank, p = 0.019; n = 60 and p < 0.001; n = 62), which is in line with previous findings [19]. Combined expression of mEGFR/COX-2 was significantly associated with unfavorable overall survival in all carcinomas (p < 0.001) and the serous subtype (p = 0.006). In addition, combined mEGFR/COX-2 expression was an independent prognostic factor in multivariate analysis (HR 16.5, CI 2.6-104.7, p = 0.003) adjusted for other prognostic factors (Table 5). Combined mEGFR/CRM1 showed a trend for unfavorable overall survival in all carcinomas (HR 3.3, CI 0.9-11.7, p = 0.06). The combination of EGFR/COX-2/CRM1 had no significant impact on overall survival (HR 3.0, CI 0.9-10.6, p = 0.8).

**Table 5:** Multivariate logistic regression analysis

Characteristic	Hazard Ratio (95%CI)	p-value
Combined mEGFR/COX-2 (pos. vs. neg.)	16.5 (2.6-104.7)	0.003
Age	1,1 (1.0-1.2)	0.008
FIGO stage (I+II vs. III+IV)	6.3 (0.6-60.9)	0.11
Tumor grade (G1 vs. G2+3)	2.2 (0.1-36.6)	0.57
Residual tumor (< 2 vs. ≥ 2 cm)	8.9 (1.6-49.6)	0.01

### Expression of EGFR in ovarian cancer cells

**Figure 4:**
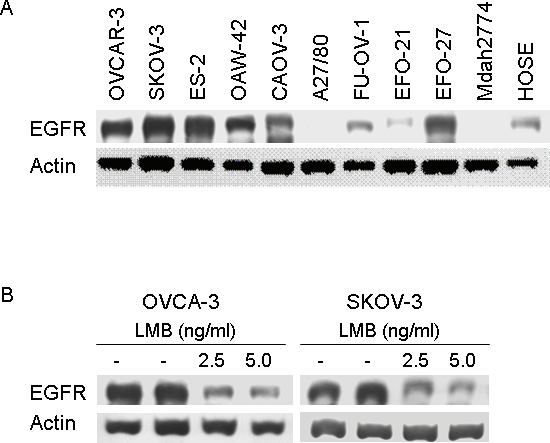
**Expression of EGFR in ovarian cancer cells and effects of Leptomycin B**. A: EGFR expression in ovarian cancer cell lines as well as human ovarian surface epithelial (HOSE) cells using western blot. B: Suppression of EGFR protein levels after exposure to Leptomycin B (LMB) in OVCAR-3 and SKOV-3 cells.

We investigated the EGFR protein expression in ten ovarian cancer cell lines as well as in immortalized human ovarian surface epithelium cells (HOSE) by Western Blot. We observed high EGFR levels in several ovarian cancer cells in particular in OVCAR-3, SKOV-3, ES-2, OAW-42, CaOV-3, and EFO-27 cells. In contrast, A27/80 and Mdah2774 showed no expression of EGFR. The FU-OV-1 and EFO-21 cancer cells as well as HOSE cells showed only weak expression (Figure 4A).

### Suppression of EGFR after exposure to Leptomycin B (LMB)

Due to the association of CRM1 and EGFR protein expression in ovarian carcinomas, we aimed to evaluate the interaction of both in a cell culture model. For this purpose, we treated two ovarian cancer cell lines with Leptomycin B (LMB), which specific inactivates CRM1 and thus the nuclear export of proteins. Incubation of OVCAR-3 and SKOV-3 cells with 2.5 and 5 ng/ml Leptomycin B revealed a significant suppression of EGFR protein levels (Figure 4B).

## Discussion

In this study, we used a novel antibody to assess the EGFR protein expression in primary invasive ovarian carcinomas. We observed a membranous EGFR expression in 36.4% which was an independent predictor of factor for poor overall survival in ovarian cancer patients.

There are several reports on EGFR as an adverse prognostic indicator in different tumor types. In ovarian carcinomas different results exist concerning EGFR expression and disease outcome. Psyrri et al. reported on an association of EGFR protein expression with poor disease-free and overall survival in a cohort of 81 advanced ovarian carcinomas (FIGO III/IV) with intraoperative residual disease more than 2 cm in 75% of cases [20]. In another cohort of 379 serous ovarian carcinomas amplification and membranous protein over-expression of EGFR were also related to poor overall survival [5]. In contrast, de Graeff et al. did not observe any association between membrane EGFR expression assessed by immunostaining and disease outcome in a prospective study of 232 ovarian carcinomas [21]. Similarly, Nielsen et al. reported on an EGFR over-expression in 62% of 783 ovarian cancer patients which had no prognostic impact [8]. As reviewed in a meta-analysis of de Graeff et al., reported EGFR expression is ranging from 6 to up 70% in ovarian carcinomas [3]. The variability among the studies is most likely caused by different assessment methods and antibodies, scoring systems, standardization approaches, and different study cohorts. In this project, we used an antibody which detects the intracellular domain of the receptor, whereas previous studies applied an antibody detecting the external receptor domain [4,5,7].

We found no association with standard clinico-pathological factors which is in line with findings from other authors [20]. In contrast, other reports showed an association of EGFR expression with higher patient age, larger residual tumor size [5], and high-grade serous carcinomas [5,22], while de Graeff et al. found that over-expression of EGFR was more frequent in non-serous tumors [21].

In this study, we demonstrate for the first time an association between EGFR and the nuclear export protein CRM1 in ovarian cancer tissue. CRM1 is responsible for nucleo-cytoplasmic shuttling of mRNAs and proteins of cancer related molecules including ErbB2 [12]. Due to the *in vivo *findings, we further aimed to investigate the interaction of EGFR and CRM1 in a cell culture model. The reduced EGFR protein expression levels after exposure with Leptomycin B in ovarian cancer cells suggest a role of CRM1 in the intracellular transactivation of EGFR. In contrast, Lo et al. detected increased nuclear EGFR levels following LMB incubation for a maximum of 4 hours in A431 human epidermoid carcinoma cells [13]. The intracellular transit and abidance in specific cell compartments of EGFR may depend on the cell type, stimulation with growth factors, and duration of CRM1 inhibition.

In our analysis, we also observed an association between membranous EGFR and COX-2. Several studies have reported a crosstalk between EGFR and COX-2. Xu et al. reported that EGF-mediated stimulation of EGFR in human glioma cell lines induces expression of both COX-2 mRNA and protein [23]. As reviewed by Dannenberg et al., EGFR signaling may lead to AP-1-mediated induction of COX-2 transcription via increased MAPK activity. In turn, enhanced prostanoid production driven by COX-2 can activate EGFR signaling [24]. Moreover, Jeong KJ and colleagues demonstrated that lysophosphatidic acid receptor, LPA2 and Gi/Src transactivation to EGFR are responsible for COX-2 expression in ovarian cancer cells [25]. The interaction of EGFR and COX-2 might be relevant for combined treatment strategies. Gupta et al. used genetic and pharmacological agents for inactivation of four genes, as the EGFR ligand epiregulin, COX-2, and two metalloproteinases (MMP1 and 2) and demonstrated a repression of primary tumor growth, tumor cell intra- und extravasation as well as metastatic outgrowth [26]. So far, only few studies have explored the possibility of an interaction of both molecules in ovarian cancer. In two immunohistochemical studies no association was found [27].

These data and our findings show a possible interaction of EGFR, COX-2, and CRM1 in ovarian cancer. In our previous studies, COX-2 was identified as an independent prognostic factor for poor overall survival in ovarian cancer patients [19] and related to CRM1 in vitro and in vivo [14]. The prognostic impact as well as its correlation and regulation of each other suggest an important role in the progression of ovarian cancer that has to be confirmed in further studies. Moreover, this crosstalk is interesting for combination therapies in anti-cancer treatment. So far, the application of anti-EGFR therapy in ovarian cancer has been shown only limited efficacy; therefore further studies will be needed to investigate whether (membranous) EGFR is also predictive for benefit from EGFR targeted therapies.

## Conclusions

In this study, we show that membranous EGFR expression is an independent prognostic factor for poor overall survival in ovarian cancer patients. We further demonstrate a significant association of EGFR with COX-2 and nuclear CRM1 expression. In addition, combined membranous EGFR/COX-2 expression is significantly related to unfavorable overall survival. In cell culture, we show a suppression of EGFR protein levels after exposure to the CRM1 inhibitor Leptomycin B in OVCAR-3 and SKOV-3 cells. Our results suggest that the EGFR/COX-2/CRM1 interaction might be involved in progression of ovarian cancer and patient prognosis. Hence, it is an interesting anti-cancer target for a combination therapy. Further studies will also be needed to investigate whether EGFR is also predictive for benefit from EGFR targeted therapies.

## Competing interests

The authors declare that they have no competing interests.

## Authors' contributions

Conception and design: AN, CD, WW. Provision of study material: JS, EIB. Collection and assembly of data: AN, CD, WW, MS, ACB, SDE, JS, EIB, MD. Data analysis and interpretation: MS, AN, CD, WW, JB. Manuscript writing: AN, CD, WW, SDE, JS. All authors read and approved the final manuscript.

## Pre-publication history

The pre-publication history for this paper can be accessed here:

http://www.biomedcentral.com/1471-2407/11/294/prepub
